# Structural phase transitions and photoluminescence properties of oxonitridosilicate phosphors under high hydrostatic pressure

**DOI:** 10.1038/srep34010

**Published:** 2016-10-13

**Authors:** Agata Lazarowska, Sebastian Mahlik, Marek Grinberg, Guogang Li, Ru-Shi Liu

**Affiliations:** 1 Institute of Experimental Physics, Faculty of Mathematics, Physics and Informatics, University of Gdansk, WitaStwosza 57, 80–308 Gdansk, Poland; 2 Department of Chemistry, National Taiwan University, Taipei 106, Taiwan; 3 Faculty of Materials Science and Chemistry, China University of Geosciences, Wuhan 430074, China; 4 Department of Mechanical Engineering and Graduate Institute of Manufacturing Technology, National Taipei University of Technology, Taipei 106, Taiwan

## Abstract

Spectroscopic properties of a series of (Sr_0.98-x_Ba_x_Eu_0.02_)Si_2_O_2_N_2_ (0 ≤ x ≤ 0.98) compounds has been studied under high hydrostatic pressure applied in a diamond anvil cell up to 200 kbar. At ambient pressure the crystal structures of (Sr_0.98-x_Ba_x_Eu_0.02_)Si_2_O_2_N_2_ (0 ≤ x ≤ 0.98) are related to the ratio of strontium to barium and three different phases exists: orthorhombic *Pbcn*(0.78 ≤ x ≤ 0.98), triclinic *P1* (0 < x ≤ 0.65) and triclinic *P1* (0.65 < x < 0.78). It was found that Eu^2+^ luminescence reveals abrupt changes under pressure (decay time, energy and shape) which indicate the variation of the local symmetry and crystal field strength in Eu^2+^ sites. These changes are attributed to the reversible pressure-induced structural phase transitions of triclinic (Sr_0.98-x_Ba_x_Eu_0.02_)Si_2_O_2_N_2_ into orthorhombic structure. Pressure in which phase transition occurs decreases linearly with increasing of Ba composition in (Sr_0.98-x_Ba_x_Eu_0.02_)Si_2_O_2_N_2_ series. Additionally, very different pressure shifts of the Eu^2+^ luminescence in different phases of (Sr0.98-xBaxEu0.02)Si_2_O_2_N_2_:Eu from −40 cm^−1^/kbar to 0 cm^−1^/kbar have been observed. This effect is explained by different interaction of the Eu^2+^ 5d electron with the second coordination sphere around the impurity cations.

Rare earth activated oxonitridosilicate compounds have been widely explored as advanced phosphors in white emitting diodes (WLEDs) due to very efficient and thermally stable luminescence that covers a broad spectral range, under excitation of blue and/or UV light^1^,^2^,^3^,^4^,^5^. Moreover, since these materials have been synthesized under ambient pressure they have a lower production cost than high-pressure synthesized nitridosilicates materials such as M_2_Si_5_N_8_:Eu (M = Ca, Sr, Ba), MAlSiN_3_:Eu (M = Ca, Sr)^5^,^6^,^7^,^8^,^9^,^10^. Among oxonitridosilicate phosphors, there is an increasing interest in layered MSi_2_O_2_N_2_:Eu (M = Ca, Sr, Ba) which show efficient yellow, green and cyan emission, respectively^11^,^12^,^13^,^14^,^15^,^16^,^17^. The present research in rare-earth ions doped MSi_2_O_2_N_2_ (M = Ca, Sr, Ba) mainly focuses on the development of an appropriate host composition by mixing alkaline earth metal cations to adjust their emission properties. For example, structure variations in MSi_2_O_2_N_2_:Eu (M = Ba, Sr) with gradually substitution of Ba with Sr leads to continous or abrupt changes in luminescence properties of these compounds^14^,^15^,^18^,^19^.

Based on high resolution synchrotron XRD measurements, it has been shown that crystal structures of the (Sr_0.98-x_Ba_x_Eu_0.02_)Si_2_O_2_N_2_ (0 ≤ x ≤ 0.98) are related to the ratio of strontium to barium. Depending on barium content three different phases exist: *Phase A* (0.78 ≤ x ≤ 0.98), *Phase B* (0 < x ≤ 0.65) and *Phase C* (0.65 < x < 0.78)^18^. *Phase A* and *Phase B* are isostructural to well-known crystal structures of BaSi_2_O_2_N_2_ and SrSi_2_O_2_N_2_, respectively, whereas *Phase C* adopts a distorted variation of the BaSi_2_O_2_N_2_ type structure^14^. The crystal structure of *Phase A* with orthorhombic unit cell is described by Pbcn space group, while *Phase B* with a triclinic unit cell is described by P1 space group. *Phase C* is a new crystal structure that is different from *Phase A* and *Phase B* and can be regarded as a “transition state” between them, since it combines the unit cell-metrics of the *Phase A* type with corrugated metal-ion layers found in the *Phase B*. Nominally *Phase C* with triclinic unit cell is attributed to P1 space group. Detailed studies on the high resolution transmission electron microscopy and scanning electron diffraction analysis of selected areas, have shown that *Phase C* usually contains a small amount of nanocrystalline domains of *Phase B* with a triclinic unit cell^18^,^19^. This could be due to inhomogenous distribution of Ba and Sr components in the mixed (Sr_0.98-x_Ba_x_)Si_2_O_2_N_2_ crystals. In (Sr0.98-xBaxEu0.02)Si_2_O_2_N_2_, the barium, strontium and europium ions are in the channels, which are formed by [SiON_3_] tetrahedra and forms linear chains in the structure. In the case of *Phase A* and *Phase C* structures, cation chains are located in a small distance from each other, which is almost equal to the intra-chain distance forming a plane in the structure, while in the case of *Phase B* cation chains are separated from each other much more than the intra-chain distance. Thus the second coordination sphere around Eu^2+^ ions in *Phase A* and *Phase C* consist of Sr^2+^/Ba^2+^ cations forming rectangle while in the case of *Phase B* the Sr^2+^/Ba^2+^ cations form a line. Cation distribution in the second coordination sphere can results in a very different spectroscopic properties of Eu^2+^ luminescence. Poort *et al.*^20^ have suggested that electron–lattice interaction energy, manifested by large Stokes shift between emission and absorption spectra is large when Eu^2+^ ions occupy the lattice sites that belongs to the linear cationic chain.

The high pressure study of the (Sr_0.98-x_Ba_x_Eu_0.02_)Si_2_O_2_N_2_ doped with Eu^2+^ presented in this contribution is continuation and extension of the research work presented in ref. 18. where the relations between the luminescence properties and the content of Ba and Sr have been discussed.

It is well known that pressure is an important physical parameter that can induce the structural phase transitions^21^,^22^. X-ray diffraction study of (Sr_0.98_Eu_0.02_)Si_2_O_2_N_2_ under hydrostatic pressure has shown that in pressure range up to 96.5 kbar triclinic structure was conserved^23^. The main motivation on performing high hydrostatic pressure was to investigate whether high pressure influences the structural stability of (Sr_0.98-x_Ba_x_Eu_0.02_)Si_2_O_2_N_2_ (0 < x ≤ 0.98) to induce phase transitions. Phase transitions were identified trough a comparison of the luminescence and Raman properties under pressure. High pressure, which permits us to continuously vary structural parameters, allow to answer question whether phase transitions are related to structural parameters like bond length and lattice constants. To achieve that, complementary studies of chemical pressure (through replacement of Sr^2+^ by larger Ba^2+^) and external high hydrostatic pressure up to 200 kbar (20 GPa) applied in diamond anvil cell (DAC) have been carried out. Finally, we have developed the phase diagram of (Sr_0.98-x_Ba_x_Eu_0.02_)Si_2_O_2_N_2_ which shows the relation between chemical and external pressure compression effects.

Properties of complex consisting of Eu^2+^ ions and its negatively charged coordination sphere can be described using crystal field approximation in such case high hydrostatic pressure causes the increase of the energies of the 4f ^7^, 4f ^6^5d^1^ electronic configuration with respect to the host bands energies and the increase of the 4f ^6^5d^1^ levels splitting^24^,^25^. The superposition of these effects are seen as a pressure-induced diminishing of the energy of luminescence related to the 4f ^6^5d^1^ → 4f ^7^ transition. The influence of the second coordination sphere may bring about drastic changes in this effect, since it can magnify or cancel the influence of the first coordination sphere. As a result, different pressure behavior of the luminescence band related to the 4f ^6^5d → 4f ^7^ transition of Eu^2+^ ions in different phases is expected.

The study of luminescence under high hydrostatic pressure results in better understand the relationship between pressure and crystal phases of oxyntridosilicate phosphors, and further clarify the influences of these phase transitions on photoluminescence properties, which might be general to oxynitride materials and will be useful in tuning optical and other properties that are sensitive to local coordination environments.

## Results

For spectroscopic studies the representatives of three different phases were selected: (Ba_0.98_Eu_0.02_)Si_2_O_2_N_2_ *(Phase A; orthorhombic; Pbcn)*, (Sr_0.49_Ba_0.49_Eu_0.02_)Si_2_N_2_O_2_ *(Phase B; triclinic; P1)* and (Sr_0.23_Ba_0.75_Eu_0.02_)Si_2_O_2_N_2_ *(Phase C; triclinic; P1)*.

**Figure 1:**
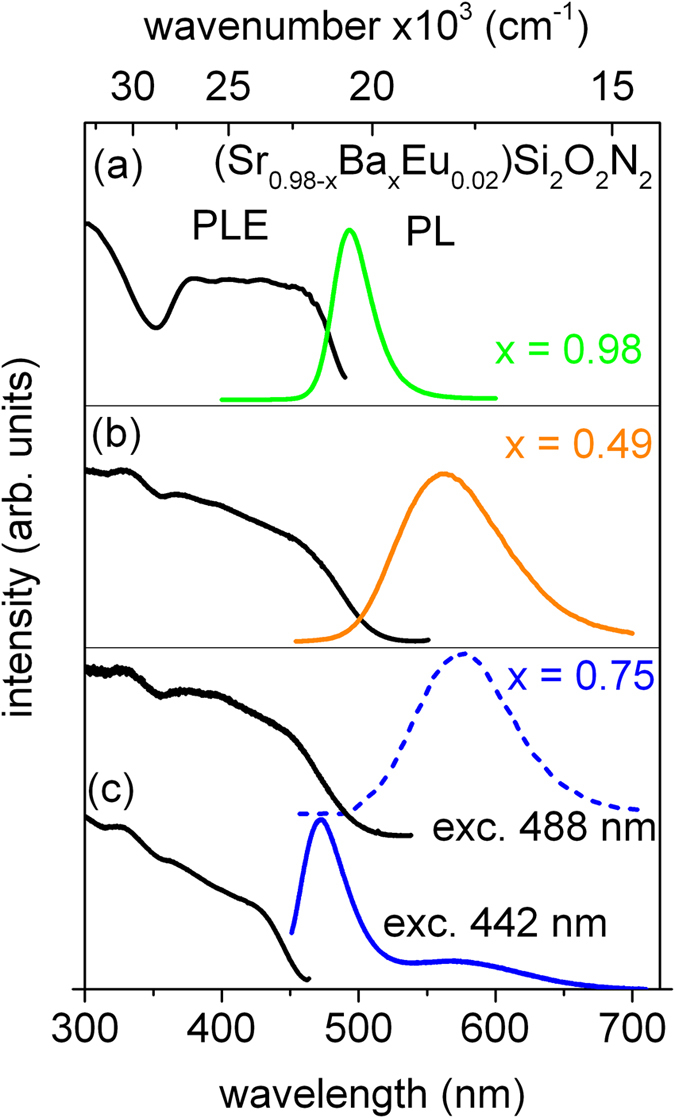
PLE and PL spectra of (Sr_0.98-x_Ba_x_Eu_0.02_)Si_2_O_2_N_2_ samples (**a**) x = 0.98 (**b**) x = 0.49 (**c**) x = 0.75. Emission spectra are excited at 442 nm (and additionally at 488 nm for x = 0.75). Excitation spectra were monitored at maximum of luminescence intensity.

**Figure 2:**
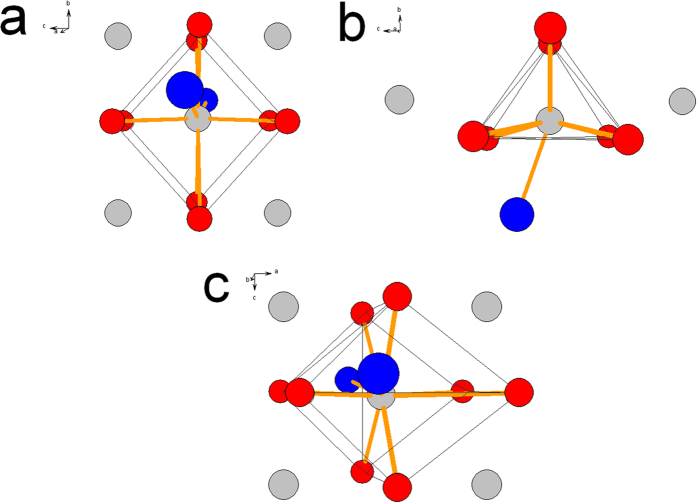
The first and second coordination of Eu^2+^ central ions in three phases of (Sr_0.98-x_Ba_x_Eu_0.02_) Si_2_O_2_N_2_ samples (**a**) x = 0.98 (*Phase A*; orthrorhombic; Pbcn), (**b**) x = 0.49 (*Phase B*; triclinic; P1) (**c**) x = 0.75 (*Phase C*; triclinic; P1). Eu^2+^/Sr^2+^/Ba^2+^-ions are drawn in light grey spheres, O-ions in red spheres and N-ions in blue spheres.

Photoluminescence excitation (PLE) and photoluminescence (PL) spectra of the (Sr_0.98-x_Ba_x_Eu_0.02_)Si_2_O_2_N_2_ system for x = 0.98 (*Phase A*) x = 0.49 (*Phase B*) and x = 0.75 (*Phase C*) are presented in Fig. 1a–c, respectively. To interpret the spectra the first and the second coordination sphere in the vicinity of Eu^2+^ ions in different crystal phases were considered. The first and the second coordination of Eu^2+^ in Sr^2+^/Ba^2+^ sites in *Phase A*, *B* and *C* are presented in Fig. 2a–c, respectively.

PLE spectra consist of several bands related to the parity allowed transitions from the ground state 4f ^7^(^8^S_7/2_) to excited states of the 4f ^6^5d^1^ electronic configuration of Eu^2+^ ions. The PL spectra consist of broad band(s) which is (are) related to the 4f ^6^5d^1^ → 4f ^7^ (d-f) transition of Eu^2+^ ions. The maximum of luminescence band varies from blue to green spectral region, depending on the ratio of Ba^2+^ to Sr^2+^. The PL spectrum of the (Ba_0.98_Eu_0.02_)Si_2_O_2_N_2_ consists of a single band peaking at 495 nm with a full width at half maximum (FWHM) equal to 32 nm (see Fig. 1a). In the orthorhombic phase (space group Pbcn, *Phase A*)^18^ of (Ba_0.98_Eu_0.02_)Si_2_O_2_N_2_, Eu^2+^ ions can occupy Ba^2+^ site which is coordinated by eight oxygen ions creating cuboid and two long ranged nitrogen ions (see Fig. 2a)^13^,^14^. The Ba-O bond lengths range from 2.74 Å to 3.10 Å and the average distance is equal to 2.89 Å whilst Ba-N distances are equal for booth nitrogen and the distance is equal to 3.31 Å.

We consider this to be the first coordination sphere. The Ba^2+^ ions form linear chains in the lattice. The distance between two separate Ba^2+^ chain is almost equal to distance between Ba^2+^ ions in a given chain, thus the second coordination sphere consist of an Ba^2+^ cations forming rectangle around Eu^2+^ central ion (see Fig. 2a).

The PL spectrum of the (Sr_0.49_Ba_0.49_Eu_0.02_)Si_2_N_2_O_2_ is presented in Fig. 1b. The spectrum consists of a broad band with a maximum at 560 nm and FWHM equal to 80 nm. In the triclinic phase (space group *P1, Phase B*)^12^,^14^,^16^ of the (Sr_0.49_Ba_0.49_Eu_0.02_)Si_2_N_2_O_2_there are four different Sr^2+^/Ba^2+^ crystallographic sites, which can be occupy by Eu^2+^ ions. These four sites are very similar, and have nearly the same surrounding. Each of them can be depicted as a distorted trigonal prism of six oxygen ions around Sr^2+^/Ba^2+^ which is capped by one N ion (see Fig. 2b)^12^,^16^. The Sr/Ba-O bond lengths range from 2.50 Å to 3.05 Å (the average distance is equal to 2.67 Å) and Sr/Ba-N distance is equal to 2.58 Å.

The Sr^2+^/Ba^2+^ ions in this case are in channels, which forms almost linear chains and the distance between two separate Ba^2+^/Sr^2+^ chains are longer than between Ba^2+^/Sr^2+^ ions in a given chain. Thus the second coordination sphere around central ions consist of two Sr^2+^/Ba^2+^ cations forming the line (see Fig. 2b). When Eu^2+^ ion is built into this chain the attractive potential of the close positive charges orients and elongates the 5d orbital of Eu^2+^ into the chain direction partially out of the first coordination sphere^20^. As the result, the 5d electron is more delocalized than in the case of *Phase A,* where no preferential orientation and elongation of 5d orbital occurs. Such delocalization of the 5d^1^ electron in Eu^2+^ is responsible for the fact that the energy of electron-lattice interaction in the excited state of Eu^2+^ is larger than in *Phase A* and results in a lower energy of the luminescence band and larger FWHM.

The PL spectra of the (Sr_0.23_Ba_0.75_Eu_0.02_)Si_2_O_2_N_2_ obtained with different excitation wavelength are presented in Fig. 1c. Under UV to blue excitation, the emission spectrum consists of two broad bands (solid curve). The first band peaking at 470 nm with FWHM equal to 41 nm is attributed to the d-f emission of Eu^2+^ occupying Sr^2+^/Ba^2+^ positions in *Phase C (triclinic P1)*. In this phase there is one Ba^2+^/Sr^2+^ site, which can be depicted as a distorted cuboid of eight O^2−^ ions (mean distance between oxygen ions and central ion is equal to 2.92 Å) and two long-ranged N^3−^ ions 3.14 Å and 3.57 Å.

It is worth noting that among these three environments of Eu^2+^ ions (*Phase A, B, C*) the average distance between the Eu^2+^ ion and nearest O/N ions (first coordination sphere) is the largest in *Phase C and smallest in Phase B*. Similar but smaller is in the phase A. The differences in the average distances result in the differences in the crystal field splitting of the 4f ^6^5d electronic manifold of Eu^2+^ ion. The smallest crystal field splitting hence the maximum of Eu^2+^ luminescence has the highest energy in *Phase C*. The strong shift of Eu^2+^ luminescence into red in *Phase B* is the result of the largest crystal field splitting.

The second coordination sphere around central ions consists of the Ba^2+^ cations forming rectangle around central ion (see Fig. 2c). This situation is similar to the case of *Phase A,* and there is no preferential orientation in the space and elongation of d orbitals, thus the Eu^2+^ luminescence is observed at high energy with a small FWHM. The second band in the yellow range of the spectrum is attributed to the occurrence of a small amount of nanocrystalline domains that contains less Ba than x = 0.75 (domains of *Phase B*). This could be due to inhomogenous distribution of a Ba and Sr components in the mixed (Sr_0.98-x_Ba_x_)Si_2_O_2_N_2_ crystals.

Emission spectra related to Eu^2+^ in domains of *Phase B* were obtained when the (Sr_0.23_Ba_0.75_Eu_0.02_)Si_2_O_2_N_2_ was excited with wavelength 488 nm (dotted curve in Fig. 1c). This emission consists of a broad band peaked at 575 nm with FWHM equal to 83 nm. Position of the maximum of the luminescence band allows to relate this emission to the *Phase B* domains of (Sr0.98-xBaxEu0.02)Si*2*O*2*N*2* with concentration x = 0.63^18^.

**Figure 3:**
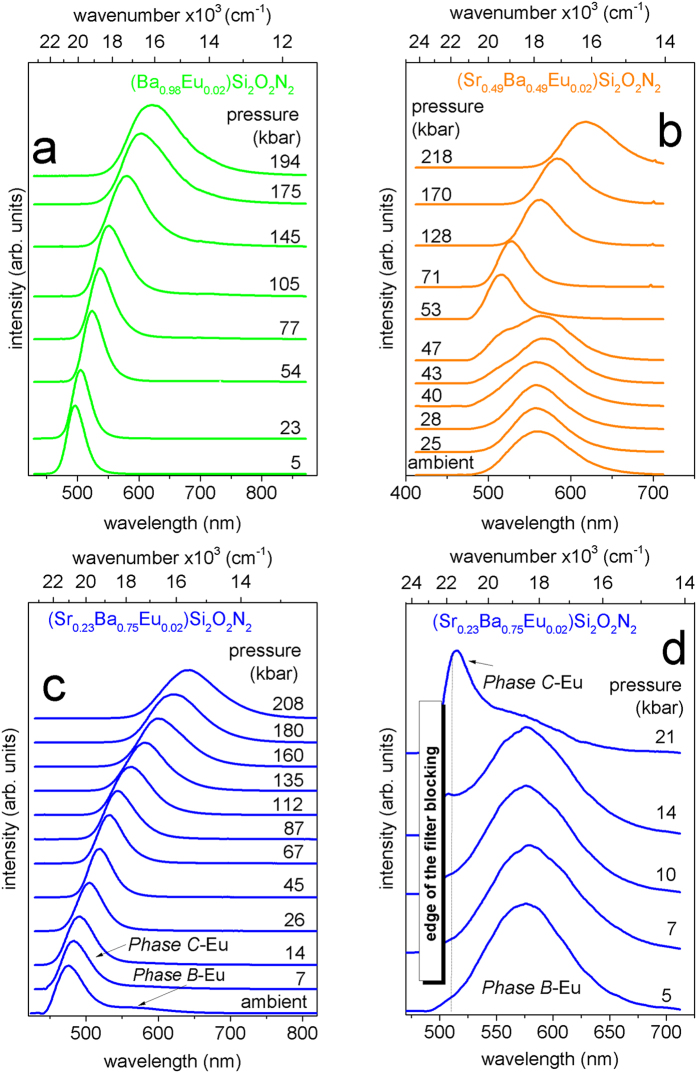
PL spectra of (Sr_0.98-x_Ba_x_Eu_0.02_)Si_2_O_2_N_2_ under different pressures (**a**) x = 0.98 excited at 442 nm (**d**), x = 0.49 excited at 442 nm, (**c,d**) x = 0.75 excited at 442 nm and 488 nm, respectively.

**Figure 4:**
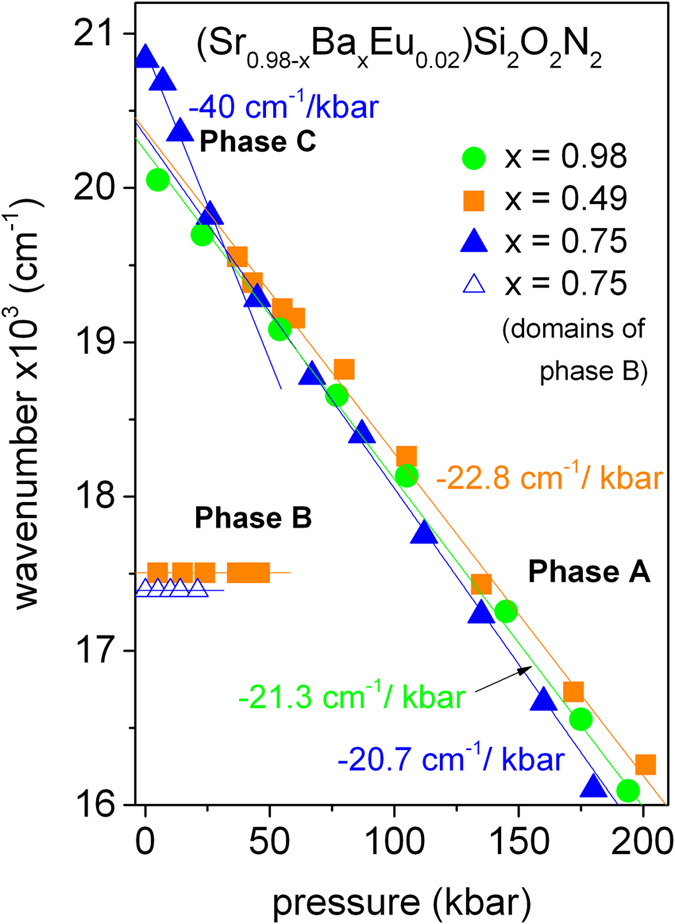
Pressure dependence of the maximum of PL spectrum (Sr_0.98-x_Ba_x_Eu_0.02_)Si_2_O2N2 for x = 0.98 –green solid circles, x = 0.49 –orange solid squares and x = 0.75 –blue solid and open triangles.

Photoluminescence spectra of the (Sr0.98-xBaxEu0.02)Si*2*O*2*N*2* (x = 0.98, 0.49, 0.75) measured at different high hydrostatic pressures are presented in Fig. 3(a–d). Energies of the (Sr0.98-xBaxEu0.02)Si*2*O*2*N*2*(x = 0.98, 0.49 and 0.75) emission maxima versus pressure are presented in Fig. 4. In Fig. 4 the green solid circles refers to the (Ba_0.98_Eu_0.02_)Si_2_O_2_N_2_, the orange squares refers to the (Sr_0.49_Ba_0.49_Eu_0.02_)Si_2_O_2_N_2_ and solid and open blue triangles refer to the (Sr_0.23_Ba_0.75_Eu_0.02_)Si_2_O_2_N_2_ samples.

In the case of the (Ba_0.98_Eu_0.02_)Si_2_O_2_N_2_ (*Phase A*) a red shift of the 4f ^6^5d^1^ −4f ^7^ emission band is observed (see Fig. 3a). The energy of the emission maximum shifts linearly with increasing pressure with a rate of about-20 cm^−1^/kbar (see Fig. 4, green circles).

Figures 3(b) and 4 (orange solid squares) present the pressure evolution of the (Sr_0.49_Ba_0.49_Eu_0.02_)Si_2_O_2_N_2_ luminescence. Up to 40 kbar the PL spectrum with maximum at 560 nm does not change. At pressures above 40 kbar additional luminescence band with maximum at about 500 nm appears. The band with maximum at 560 nm is quenched with increasing pressure and vanishes at 50 kbar. Above 50 kbar only the band with maximum at about 500 nm is seen. Further increase of pressure up to 200 kbar causes the shift of emission maximum toward to the lower energies with rate approximately equal to −20 cm^−1^/kbar (see Fig. 4 orange solid squares). Such behavior of the PL of the (Sr_0.49_Ba_0.49_Eu_0.02_)Si_2_O_2_N_2_ versus pressure can be attributed to the pressure induced structural transformation of the Eu^2+^-ligand system. At ambient condition the (Sr_0.49_Ba_0.49_Eu_0.02_)Si_2_O_2_N_2_ has a triclinic SrSi_2_O_2_N_2_-type structure (*Phase B*). The new emission signal that appears at pressure above 40 kbar can be related to beginning of a structural transformation of the (Sr_0.49_Ba_0.49_Eu_0.02_)Si_2_O_2_N_2_. Since from 40 to 50 kbar the PL spectrum of the (Sr_0.49_Ba_0.49_Eu_0.02_)Si_2_O_2_N_2_ consist of two bands we propose that two phases coexist in this pressure range. One notices that at 50 kbar the luminescence band of the (Sr_0.49_Ba_0.49_Eu_0.02_)Si_2_O_2_N_2_ has maximum at the same energy as the (Ba_0.98_Eu_0.02_)Si_2_O_2_N_2_. Moreover, when pressure increases above 50 kbar the luminescence maximum shifts with the same rate (−22.8 cm^−1^/kbar–orange solid squares) as it has been observed for (Ba_0.98_Eu_0.02_)Si_2_O_2_N_2_ (−21.3 cm^−1^/kbar-green solid circles). This indicates that pressure-induced phase transition from triclinic (Sr_0.49_Ba_0.49_Eu_0.02_)Si_2_O_2_N_2_ (*Phase B*) to orthorhombic phase (*Phase A*) takes place.

The PL spectra of the (Sr_0.23_Ba_0.75_Eu_0.02_)Si_2_O_2_N_2_ under different pressure are presented in Fig. 3(c,d). In Fig. 3(c) the 4f 65d1 − 4f 7Eu^2+^ emission, obtained under excitation with wavelength 442 nm is presented. When pressure increases up to 20 kbar, energy of the maximum of emission band decreases (the red shift is approximately equal to −40 cm^−1^/kbar). Above 20 kbar, the red shift rate diminishes and is approximately equal to −20 cm^−1^/kbar (see solid blue triangles in Fig. 4) which is similar to that observed for orthorhombic (Ba_0.98_Eu_0.02_)Si_2_O_2_N_2_ and high pressure orthorhombic (Sr_0.49_Ba_0.49_Eu_0.02_)Si_2_O_2_N_2_ structures. We propose that this change in the rate of the PL pressure shift, is the result of phase transition from triclinic (*Phase C*) to orthorhombic (*Phase A*) phase of the (Sr_0.23_Ba_0.75_Eu_0.02_)Si_2_O_2_N_2_ system.

Figure 3(d) presents pressure evolution of the emission of the (Sr_0.23_Ba_0.75_Eu_0.02_)Si_2_O_2_N_2_ in *Phase B* domains, excited with wavelength 488 nm. It is expected that these domains would transform into *Phase A* under pressure and this actually happened (see Figs 3d and 4). Within the pressure range from ambient to 20 kbar the PL maximum does not change and the luminescence intensity gradually decrease. Above 20 kbar this luminescence is completely quenched. The phase transition in this case occurs for a lower pressure (approximately 20 kbar) than it is observed for (Sr_0.49_Ba_0.49_Eu_0.02_)Si_2_O_2_N_2_ (approximately 50 kbar) as the ratio of Ba to Sr in the domains is higher (x = 0.63).

**Figure 5:**
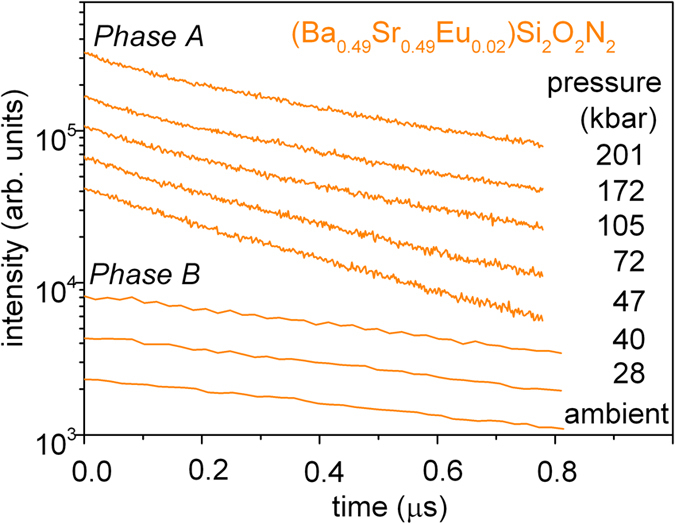
PL decays of the (Sr_0.49_Ba_0.49_Eu_0.02_)Si_2_O2N_2_ for different pressures collected at maximum of luminescence under excitation of 442 nm.

**Figure 6:**
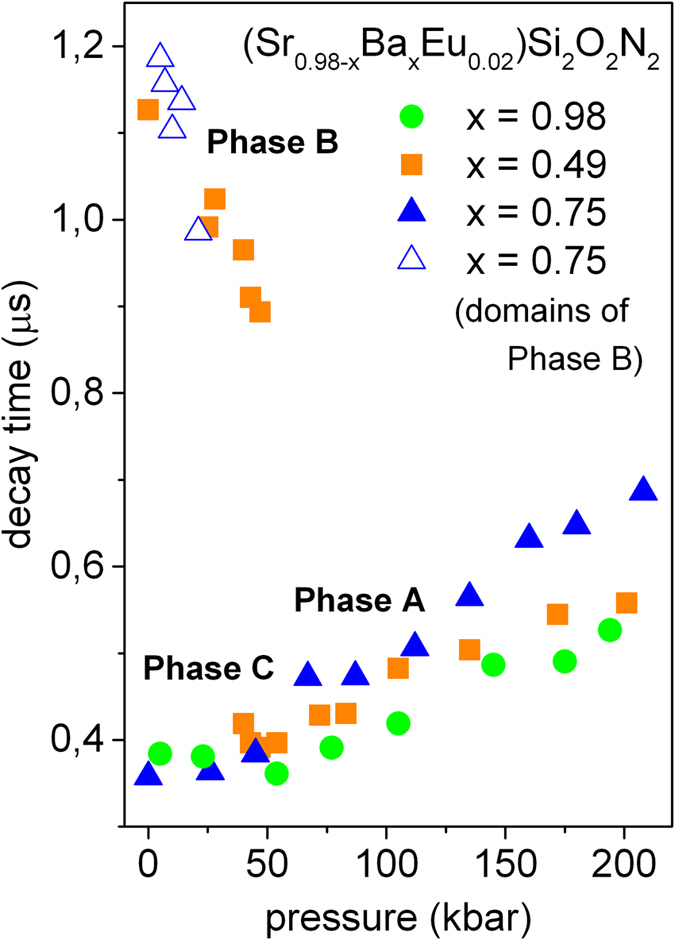
Pressure dependence of the PL decay times of (Sr_0.98-x_Ba_x_Eu_0.02_)Si_2_O2N_2_ for x = 0.98 –green circles, x = 0.49 –orange squares and x = 0.75 –open and solid blue triangles.

Room temperature luminescence decays of (Sr_0.98-x_Ba_x_Eu_0.02_)Si_2_O_2_N_2_ for x = 0.98 (*Phase A*), x = 0.49 (*Phase B*) and x = 0.75 (*Phase C* and domains of *Phase B*) recorded at different pressures were monitored at the maximum of the 5d^1^4f ^6^ → 4f ^7^transition. The example of the PL decays for the (Sr_0.49_Ba_0.49_Eu_0.02_)Si_2_O_2_N_2_ are shown in Fig. 5. All decays related to the Eu^2+^ emission in compounds under study were close to single exponential. The decay times versus pressure for all considered samples are collected in Fig. 6. The PL decay time obtained for the (Ba_0.98_Eu_0.02_)Si_2_O_2_N_2_ (green circles), at ambient pressure is equal to 0.38 μs and increase gradually to 0.54 μs at 200 kbar. In the absence of nonradiative processes this behavior can be expected because the luminescence lifetime elongates as the emission is red shifted with increasing pressure^26^.

The PL decay time obtained for the (Sr_0.49_Ba_0.49_Eu_0.02_)Si_2_O_2_N_2_ (orange squares) at ambient pressure is equal to 1.13 μs. The decay time slightly decrease with increasing pressure to 0.89 μs at 50 kbar which is accompanied by the decrease of emission intensity and at 50 kbar luminescence with maximum at 560 nm vanishes completely.

Across the phase transition at about 40 kbar, new luminescence band appeared (see Fig. 3b) and this emission decays with the time equal to 0.42 μs. Increase of pressure slightly elongates the luminescence decay like it was observed for the (Ba_0.98_Eu_0.02_)Si_2_O_2_N_2_ sample. The pressure-dependence of the PL decay time for the (Sr_0.49_Ba_0.49_Eu_0.02_)Si_2_O_2_N_2_ confirms our previous consideration of the structural transformation of *Phase B* into *Phase A* induced at about 40~50 kbar. The PL decay of the (Sr_0.23_Ba_0.75_Eu_0.02_)Si_2_O_2_N_2_ (*Phase C*) monitored at maximum of luminescence is represented by closed blue solid triangles. The decay time at ambient pressure is equal to 0.36 μs. Above 20 kbar the decay time elongates with pressure and reach the value of 0.67 μs at 200 kbar. The same behavior is observed for the (Sr_0.49_Ba_0.49_Eu_0.02_)Si_2_O_2_N_2_ above 50 kbar (after phase transition) and for the (Ba_0.89_Eu_0.02_)Si_2_O_2_N_2_ in all considered pressure. Pressure dependence of the PL decay time of the *Phase B* domains in the (Sr_0.23_Ba_0.75_Eu_0.02_)Si_2_O_2_N_2_ are represented by open blue triangles. At ambient pressure *Phase B* domains have a decay time equals 1.19 μs, which is close to the value obtained for the (Sr_0.49_Ba_0.49_Eu_0.02_)Si_2_O_2_N_2_ in *Phase B*. Pressure slightly shortens the decay time to a value of 0.99 μs for 20 kbar which is accompanied by decrease of luminescence intensity. Above 20 kbar the luminescence from *Phase B* domains is not present in the emission spectra of the (Sr_0.23_Ba_0.75_Eu_0.02_)Si_2_O_2_N_2_ system.

One should discuss the phenomenon of the Eu^2+^ luminescence in *Phase B*. In contrast to *Phases A* and *C* the Eu^2+^ emission in *Phase B* consists of broad band characterized by FWHM equal to 83 nm, which decays with the decay time 1.2 μs, whereas the Eu^2+^ emission in *A* and *C* phases is represented by much sharper band, characterized by FWHM equal to 41 nm decaying with the lifetime ~0.36 μs. Additionally very different pressure shifts of the 4f ^6^5d → 4f ^7^ luminescence of Eu^2+^ in the *Phases A* and *C* (where it is equal to −20 cm^−1^/kbar and −40 cm^−1^/kbar, respectively) and in *Phase B* (where it is equal zero) have been observed. This different pressure dependences of the emission can be attributed to different interaction of the Eu^2+^ system in the 4f ^6^5d^1^ state with the second coordination sphere of positive ions. In the case of Eu^2+^ in (Sr_0.98-x_Ba_x_Eu_0.02_)Si_2_O_2_N_2_ in *Phase B*, the 5d electron of Eu^2+^ is attracted by the positively charged chain and therefore is delocalized and can be partly located out of the first coordination sphere of negative ions. This results in large Stokes shift and FWHM of the luminescence, actually much larger than in the case of the 4f ^6^5d → 4f ^7^ luminescence of Eu^2+^ in (Sr_0.98-x_Ba_x_Eu_0.02_)Si_2_O_2_N_2_ in *Phase A* and *Phase C*.

Since pressure decrease distance between Eu^2+^ion and ligand ions, it is expected that the pressure induces the red shift of the d-f luminescence due to the increase of crystal field splitting of 5d electronic manifold. This effect has been previously observed in many different compounds^27^. The first coordination sphere of the Eu^2+^ in (Sr_0.98-x_Ba_x_Eu_0.02_) Si_2_O_2_N_2_ is build with negative ions having approximately cubic symmetry. These causes the splitting of the 5d orbital into doubly degenerated the lower state (e) and triply degenerated the higher state (t)^28^. In the (Sr_0.98-x_Ba_x_Eu_0.02_)Si_2_O_2_N_2_ having *Phase A* the second coordination sphere is built with four positive ions (approximately C_4v_ symmetry) which can be considered as an octahedron without vertical ions. This configuration magnifies the splitting caused by first configurational sphere and additionally splits the double degenerated the lower state (e) into the non-degenerated a_1_ and b_1_ states. As the result large pressure shift of the luminescence related to the 4f ^6^5d → 4f ^7^ luminescence Eu^2+^ emission in *Phases A* and *C* is observed. Due to the delocalization of the 5d electron in (Sr_1-x_Ba_x_Eu_0.02_)Si_2_O_2_N_2_ in *Phase B* the energy of the lowest state of the 5d^1^4f ^6^ electronic manifold can be not sensitive to actual positions of negative ions forming the first coordination sphere and finally does not dependent on pressure.

**Figure 7:**
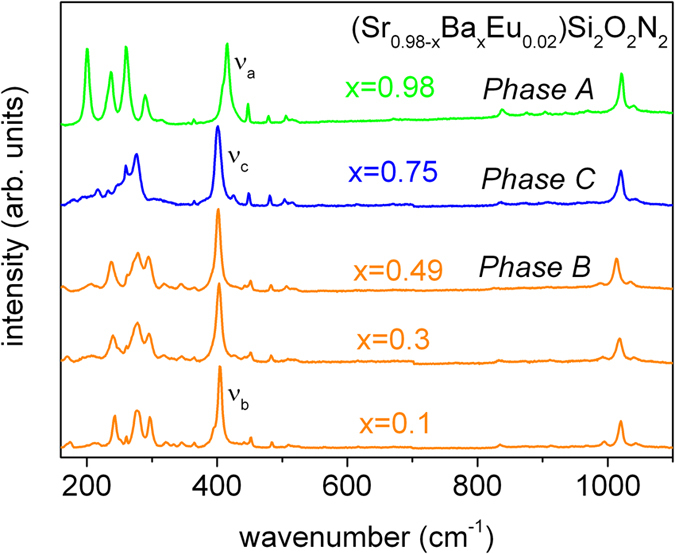
Raman spectra of (Sr_0.98-x_Ba_x_Eu_0.02_)Si_2_O_2_N2 for different Ba content x.

Raman spectroscopy has been applied to confirm structure changes of (Sr_0.98-x_Ba_x_Eu_0.02_)Si_2_O_2_N_2_ for x = 0.1, 0.3, 0.49, 0.75 and 0.98 and pressure-induced phase transition of the (Sr_0.98-x_Ba_x_Eu_0.02_)Si_2_O_2_N_2_. The ambient pressure Raman spectra of (Sr_0.98-x_Ba_x_Eu_0.02_)Si_2_O_2_N_2_ for different Ba concentrations are presented in Fig. 7. The crystal structure of oxynitridosilicates (Sr_0.98-x_Ba_x_Eu_0.02_)Si_2_O_2_N_2_ are built of SiON_3_ tetrahedral layers with the same general topology for all samples. It is expected that internal stretching and bending vibrations of the tetrahedral SiON_3_ groups contribute to Raman peaks in the range 400–1200 cm^−1^ for all samples^29^,^30^. In this spectral region, the Raman spectrum of (Sr_0.98-x_Ba_x_Eu_0.02_)Si_2_O_2_N_2_ for x = 0.98 is dominated by an intense lines with energies equal to 415 cm^−1^ (labeled ν_a_) and 1020 cm^−1^ and for x ≤ 0.75, 401~405 cm^−1^ (labeled ν_c_ and ν_b_) and 1020 cm^−1^. Difference between position of ν_a_ with respect to ν_b_ and ν_c_ peaks are due to different degree of distortion of SiON_3_ groups. The external vibrations involving the entire SiON_3_ as well as their neighboring Sr^2+^/Ba^2+^ ions are observed below 400 cm^−1 ^29,30. Here the highest intensity peaks in the spectrum of (Sr_0.98-x_Ba_x_Eu_0.02_)Si_2_O_2_N_2_ (x = 0.1) are detected at 244 cm^−1^, 261 cm^−1^, 278 cm^−1^ and 298 cm^−1^. The substitution of Sr^2+^ by Ba^2+^ up to x = 0.49 causes broadening of observed peaks but the specific Raman peak pattern is preserved. For Ba^2+^ content x = 0.75 and x = 0.98 apparently different Raman pattern is observed below 400 cm^−1^. The highest intensity peaks in the spectrum (below 400 cm^−1^) for (Sr_0.98-x_Ba_x_Eu_0.02_)Si_2_O_2_N_2_ (x = 0.75) are observed at 275 cm^−1^ and 260 cm^−1^, whereas for (Ba_0.98-x_Eu_0.02_)Si_2_O_2_N_2_ (x = 0.98) the highest intensity peaks are observed at 201 cm^−1^, 237 cm^−1^, 259 cm^−1^ and 289 cm^−1^. Difference in position and relative intensity of Raman peaks of studied samples clearly illustrate that three different phases exist across (Sr_0.98-x_Ba_x_Eu_0.02_)Si_2_O_2_N_2_ series and confirmed the previous XRD results^18^.

**Figure 8:**
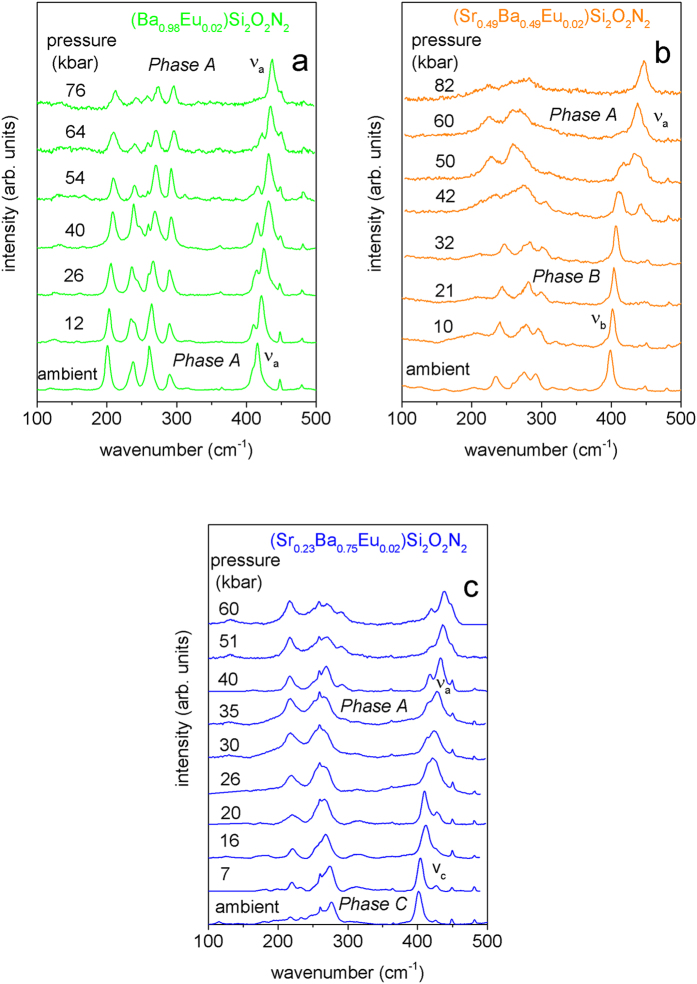
Raman spectra of the (Sr_0.98-x_Ba_x_Eu_0.02_)Si_2_O_2_N_2_ for (**a**) x = 0.98 (**b**) x = 0.49 and (**c**) x = 0.75 at different pressures.

To verify our hypothesis about pressure-induced phase transformations from triclinics (*Phase B* and C) to orthorhombic phase (*Phase A*) Raman spectra of the (Sr_0.98-x_Ba_x_Eu_0.02_)Si_2_O_2_N_2_ for x = 0.49, 0.75 and 0.98 have been measured at different pressures up to ~80 kbar and the results are presented in Fig. 8a–c. It should be noted here that in our experiments the Eu^2+^ luminescence with laser beam excitation of 632 nm contributes greatly to the background level causing the Raman spectra were very difficult or even impossible to measure under pressure greater than 80 kbar. In the case of (Ba_0.98_Eu_0.02_)Si_2_O_2_N_2_ (see Fig. 8a). It is seen that due to the decrease of bonding length, energy of all Raman peaks increases with pressure. This increase is observed with a slightly different pressure rate for all lines causing that some of unresolved peaks at ambient pressure (see for example ν_a_) start to be resolved at higher pressure, but the main Raman pattern and number of peaks does not change with pressure. These result confirms findings from luminescence studies, that the orthorhombic phase of (Ba_0.89_Eu_0.02_)Si_2_O_2_N_2_ is preserved in measured pressure range. Figure 8b shows high-pressure Raman spectra of (Sr_0.49_Ba_0.49_Eu_0.02_)Si_2_O_2_N_2_. The most interesting effect occurs above 32 kbar where a significant change in the Raman lines structure is observed. Let us focus on the most intensive vibration peak ν_b_ related to the internal stretching and bending vibrations of the SiON_3_ groups. It is seen that due to the increase of Si–O/N bonding strength energy of ν_b_ increases when pressure increases. In the range of pressure between 40 kbar and 50 kbar additional peak labeled ν_a_ appears and simultaneously intensity of ν_b_ decreases. Above 50 kbar peak ν_a_ remains exclusively. The ν_a_ Raman line can be attributed to an orthorhombic structure of the (Ba_0.98_Eu_0.02_)Si_2_O_2_N_2_. The appearance of ν_a_ peak at ~42 kbar confirms the phase transition of the (Sr_0.49_Ba_0.49_Eu_0.02_)Si_2_O_2_N_2_ from triclinic to orthorhombic phase. Figure 8c shows high-pressure Raman spectra of (Sr_0.23_Ba_0.75_Eu_0.02_)Si_2_O_2_N_2_ which is representative of *Phase C*. Also in this case we have observed significant changes in Raman spectra but at lower pressure of about 26 kbar suggesting a structural transition occurs at this point. At this pressure additional peak labeled ν_a_ appears and simultaneously intensity of ν_c_ decreases. Also obvious changes in Raman spectra below 400 cm^−1^ are observed. Upon decompression, the luminescence and structural properties of the (Sr_0.98-x_Ba_x_Eu_0.02_)Si_2_O_2_N_2_ (x = 0.98, 0.49 and 0.75) of these crystals returns to ambient state. It means that the high-pressure induced phase transition of the (Sr_0.98-x_Ba_x_Eu_0.02_)Si_2_O_2_N_2_ (0.49 and 0.75) is reversible.

**Figure 9:**
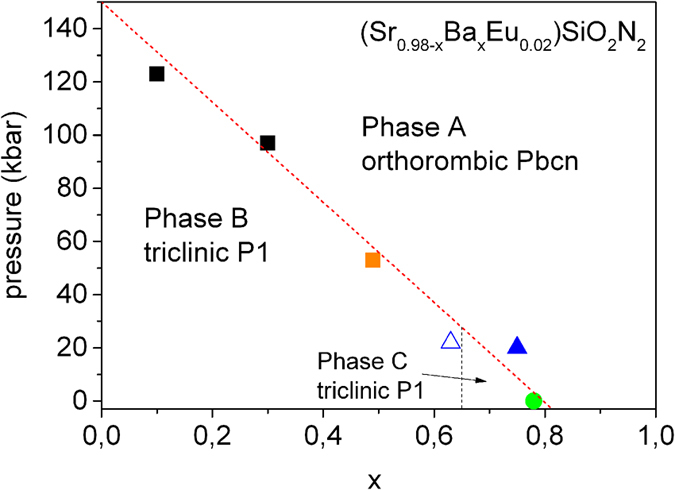
Phase diagram of (Sr_0.98-x_Ba_x_Eu_0.02_)Si_2_O_2_N_2_.

In summary we developed the phase diagram of (Sr_0.98-x_Ba_x_Eu_0.02_)Si_2_O_2_N_2_ which has been shown on Fig. 9. The dotted lines represent the phase boundaries between different phases in the pressure and Ba content space. Two points corresponding to pressures of phase transitions for (Sr_0.98-x_Ba_x_Eu_0.02_)Si_2_O_2_N_2_ x = 0.3 and x = 0.1 (black squares in Fig. 9) were obtained at high pressure luminescence experiments, however the respective luminescence and Raman spectra are not presented in this contribution. The phase diagram shows that pressure in which phase transition between *Phase B* and *A* occurs decreases linearly with increasing Ba concentration in (Sr_0.98-x_Ba_x_Eu_0.02_)Si_2_O_2_N_2_. The Boundary between *Phase B* and *A* is indicated by dotted red line. For concentration rage 0.65 < x < 0.78 and pressure lower than 20 kbar the (Sr_0.98-x_Ba_x_Eu_0.02_)Si_2_O_2_N_2_ system exists in *Phase C*. The boundary between *Phase B* and *C* is indicated by dotted black line.

## Methods

### Chemicals and Materials

SrCO_3_ (≥99.9%), SiO_2_ (≥99.995%), Si_3_N_4_ (≥99.9%), and Eu_2_O_3_ (≥99.99%) were purchased from Aldrich Corporation. BaCO_3_ (≥99.9%) was purchased from J.T. Baker Corporation. All of the initial chemicals were used without further purification. Aluminum oxide crucibles and cylindrical molybdenum crucibles (20 mm × 50 mm) were used in the sintering process of the samples.

### Synthesis

A series of oxonitridosilicate compounds, (Sr_0.98-x_Ba_x_Eu_0.02_)Si_2_O_2_N_2_ (0.1 ≤ x ≤ 0.98), was prepared using two-step solid-state reaction processes. Nonstoichiometric amounts of SrCO_3_, BaCO_3_, SiO_2_, and Eu_2_O_3_ powders, in which the molar ratio of (Sr, Ba)/Si = 0.8 were ground in an agate mortar for 30 min to form a homogeneous mixture. The mixture was then placed in aluminum oxide crucible and fired at 1250 to 1350 °C for 6 h under flowing 95%N_2_−5% H_2_ atmosphere in a horizontal tube furnace. The sintered products were ground, yielding crystalline powder (Sr_0.98-x_Ba_x_Eu_0.02_)Si_2_O_2_N_2_ (0.1 ≤ x ≤ 0.98). The crystalline powder was mixed with stoichiometric amounts of Si_3_N_4_ and reground for 30 min in an agate mortar. After forming a homogeneous mixture, the mixture was loaded into a cylindrical molybdenum crucible with a screw-cap and fired again at 1500 to 1550 °C for 6 h under flowing 95% N_2_−5%H_2_ atmosphere in the horizontal tube furnace. The second sintered products were ground again, yielding the resulting phosphor powder. The details of samples preparation and ambient pressure structural characterization were presented and discussed in paper^18^.

### Spectroscopic characterization

Raman spectra were recorded at room temperature and ambient pressure on a Horiba JobinYvon Lab Ram Aramis spectrometer with a He-Ne laser providing excitation light at 633 nm with the 1200 l/mm grating.

Photoluminescence excitation spectra were acquired using a FluoroMax-4P TCSPC spectrofluorometer produced by Horiba, containing Czerny-Turner monochromators for excitation and emission. An excitation source in this system was a 150-W ozone-free Xenon lamp. Fluorescence intensity was measured using a R928 Side-on photomultiplier. Steady state luminescence spectra were excited with He-Cd laser with the wavelength of 442 nm. The photoluminescence spectra were recorded on a SR-750-D1 luminescence spectrometer with an Andor CCD camera DU420A-OE type, which can detect signal on large wavelength range 250–1000 nm with an accuracy of 0.5 nm. The spectra were corrected for instrumental spectral response using a standard lamp.

The experimental setup for luminescence kinetics consists of a PL 2143 A/SS laser as the excitation source and a PG 401/SH parametric optical generator. This system can generate 30 ps laser pulses, with the frequency of 10 Hz with wavelengths ranging from 220 to 2200 nm. The emission signal was analyzed by a Bruker Optics 2501S spectrometer and the Hamamatsu Streak Camera model C4334-01 with a final spectral resolution 0.47 nm. Luminescence decays were obtained by the integration of streak camera images over the wavelength intervals. Details of the experimental setup are described in the paper^31^. High hydrostatic pressure was applied in a Merrill Bassett type DAC^32^. Polydimethylsiloxane oil was used as the pressure-transmitting medium, and pressure was measured by the shift of the R_1_ luminescence line of ruby.

## Additional Information

**How to cite this article**: Lazarowska, A. *et al.* Structural phase transitions and photoluminescence properties of oxonitridosilicate phosphors under high hydrostatic pressure. *Sci. Rep.* **6**, 34010; doi: 10.1038/srep34010 (2016).
